# Burden of knee osteoarthritis in China and globally from 1992 to 2021, and projections to 2030: a systematic analysis from the Global Burden of Disease Study 2021

**DOI:** 10.3389/fpubh.2025.1543180

**Published:** 2025-04-14

**Authors:** Yueming Lv, Liang Sui, Hao Lv, Jiacheng Zheng, Huichao Feng, Fujie Jing

**Affiliations:** ^1^School of Acupuncture-Tuina, Shandong University of Traditional Chinese Medicine, Jinan, China; ^2^Health Sciences, Shandong University of Traditional Chinese Medicine, Jinan, China

**Keywords:** disease burden, knee osteoarthritis, prediction study, years lived with disability, epidemiology, gender disparities

## Abstract

**Background:**

Knee osteoarthritis (KOA) is primarily characterized by joint pain and dysfunction, and KOA has increasingly emerged as a public health concern in China and globally. This study aims to utilize data from the Global Burden of Disease (GBD) 2021 to summarize the disease burden of KOA in China and globally from 1992 to 2021, while also predicting the disease burden in 2030.

**Methods:**

Using data from the GBD 2021 study, we compared and described the burden of KOA in China and globally. Joinpoint regression was applied to assess long-term trends in the burden of KOA based on GBD 2021 data. The impact of population growth, aging, and epidemiological trends on the burden of KOA was examined through decomposition analysis. Additionally, an age-period-cohort analysis (APC) was conducted to assess the effects of age, period, and cohort on the burden of KOA in China. Finally, we predicted the burden of KOA in 2030 using the Bayesian age-period-cohort (BAPC) and Norpred models.

**Results:**

In 2021, the number of patients with KOA in China was 10,957,472, reflecting an increase of 157.15% compared to 1992. Similarly, the incidence of KOA in China for the same year was 8,512,396, representing a rise of 123.45% since 1992. The and Years lived of disabled (YLDs) rate for KOA in China was 249.81 per 100,000 population, which is 116.44% higher than the rate observed in 1992. In 2021, the prevalence of KOA increased with age. Female exhibited higher estimates of prevalence, incidence, and YLDs than male across all age groups. Joinpoint regression analysis revealed fluctuating upward trends in prevalence, incidence, and YLDs, from 1992 to 2021. Decomposition analysis identified population growth as the primary driver of increased prevalence, incidence, and YLDs, particularly among female. Projections indicate that the number of KOA YLDs in China will continue to rise, potentially reaching a peak by 2030.

**Conclusion:**

The disease burden of KOA in China remains significant, necessitating increased attention, particularly for female and the middle-aged and older adult populations, in order to develop more targeted preventive measures.

## 1 Introduction

Osteoarthritis (OA) is a relatively common and disabling disease characterized by progressive damage to the articular cartilage, approximately 7.6% of the global population was affected by OA in 2020, resulting in damage to the subchondral bone and surrounding ligaments, with the knee joint being the most commonly affected site (1–4). Studies have indicated that female have a considerably higher prevalence of knee osteoarthritis (KOA) compared to male (5). KOA not only has a serious impact on the quality of life of individuals but also imposes a significant economic burden on society (6–8). A cross-sectional study from China showed that pain and dysfunction due to KOA could cause depression in older adult patients (9). Additionally, KOA creates not only a direct medical burden but also leads to loss of productivity for patients due to sick leave and absences (10, 11). During the initial phase of KOA, individuals may find relief from symptoms through exercise therapy, glucocorticoid treatment, and non-steroidal anti-inflammatory medications (12, 13). However, joint replacement surgery may be necessary in advanced stages (14). Multiple studies have shown that age, sex, and overweight are risk factors for KOA (15–17). As China’s population ages rapidly and obesity rates climb, the incidence of KOA is steadily increasing, posing a health concern that cannot be overlooked both domestically and internationally (3, 5, 18).

The Global Burden of Diseases, Injuries, and Risk Factors Study (GBD) 2021 is a worldwide epidemiological project that evaluates the incidence, prevalence, and disability-adjusted life-years (DALYs) of 371 diseases across different periods, countries, and regions (19). Although previous research has examined the burden of KOA caused by high body mass index (BMI) in China using GBD 2019 data (20), the GBD database has undergone an update, incorporating pertinent data on KOA from 2020 and 2021, thereby enhancing the data’s representativeness and currency.

Therefore, this study aims to determine the incidence, prevalence, and Years lived of disabled (YLDs) of KOA in China and globally, as well as to predict the trends of KOA using data from GBD 2021. Our ultimate goal is to refine prevention strategies and offer foundational support for shaping public health initiatives.

## 2 Methods

### 2.1 Data source

The study utilized data from the GBD 2021, providing comprehensive health metrics categorized by age, sex, and region, updated annually through international cooperation. The report details 88 risk factors, 288 causes of death, and 371 diseases and injuries across 204 nations. In China, primary data were derived from Disease Surveillance Points, censuses, population surveys, and the Chinese CDC’s Cause of Death Reporting System, and systematic reviews assessing disease incidence and prevalence (19, 21). The case definition and retrieval strategy for KOA burdens can be found in the Supplementary materials. The Global Health Data Exchange GBD Results Tool^1^ facilitated the collection of data on KOA burdens from 1992 to 2021. The database provides age-specific and age-standardized rates with 95% uncertainty intervals. In the GBD 2021, the flowchart, input data, and methodological summary for KOA burdens can be found at https://www.healthdata.org/gbd/methods-appendices-2021/osteoarthritis. Ethical approval was not required for this research since it involved publicly available, anonymized data.

### 2.2 Joinpoint

Long-term trends in the prevalence, incidence, and YLDs rates of KOA were analyzed by calculating average annual percent changes (AAPC) with 95% confidence intervals (CIs) using a joinpoint regression model (version 5.2.0; National Cancer Institute, USA). This model segments the overall trend into distinct linear segments, enabling the assessment of disease dynamics across different time periods (22). The AAPC is calculated by weighting the regression coefficients from each segment’s annual percent changes. An annual percent change greater than 0 indicates an increase in rates of KOA during that period, while an annual percent change less than 0 signifies a decrease.

### 2.3 Decomposition analysis

To identify the key drivers of the changing burden of KOA from 1992 to 2021, a decomposition analysis was utilized. This method measured the distinct impacts of epidemiological shifts, population growth, and aging (23, 24). Each factor’s impact was evaluated separately, keeping the other two factors constant.

### 2.4 Age-period-cohort modeling analysis

We conducted an age-period-cohort (APC) analysis to evaluate the influence of age, period, and cohort factors on KOA in China over the period 1992 to 2021. Data on prevalence, incidence, and YLDs were organized into five-year spans and age intervals from 30 to 94 years. Employing a log-linear model, the analysis calculated rates by aggregating influences from birth cohorts, calendar periods, and age. Age effects illustrate variance in risk across age groups; period effects capture uniform temporal shifts affecting all groups; and cohort effects reflect risk variations among peers born during the same timeframe (25–27). The R package from the Biostatistics Branch of the NIH, USA, was used for modeling and deriving estimable functions (28). For the APC analysis, we designated the median birth cohort, period group, and age group as reference categories, choosing the lower median when categories were even in number (28).

### 2.5 BAPC model projection

This study utilized the Bayesian age-period-cohort (BAPC) framework to forecast future disease burdens, leveraging its capability to handle the high-dimensional, intricate, and sparse data characteristic of large-scale epidemiological studies such as GBD 2021 (29). Rooted in the generalized linear model framework and enhanced by Bayesian techniques, the BAPC model adeptly integrates age, period, and cohort effects dynamically. Its adaptability and durability with time-series data make it particularly suitable for long-term forecasts of disease burdens. The widespread verification and use of the BAPC model in epidemiological studies, particularly those examining age-structured populations with complex cohort dynamics (30) underscore its efficacy. Utilizing the “BAPC” R package, we forecasted KOA’s burden in China using demographic projections from the IHME and GBD 2021 data, providing nuanced insights into future disease burdens by analyzing complex interplays of age, period, and cohort effects.

### 2.6 Nordpred model projection

The Nordpred model, derived from the APC framework, accurately projects future YLDs for diseases or injuries by analyzing trends and demographic shifts, including population structure changes and generational impacts (31). For estimating future trends in KOA’s burden, this model was applied to predict YLDs of KOA from 2022 to 2030.

## 3 Results

### 3.1 Trends in KOA burden in globally and China from 1992 to 2021

Globally, the prevalence of KOA in 2021 was 374,738,744 cases, an increase of 124.51% compared to 1992. Female accounted for a higher number of cases (230,189,285) than male (144,549,459). The global incidence of KOA was 30,845,891 cases in 2021, a 108.51% increase from 1992, with higher incidence in female (18,299,871 cases) than in male (12,546,020 cases). The global YLDs due to KOA reached 12,019,070 in 2021, up by 123.53% since 1992. Female experienced higher YLDs (7,344,351) compared to male (4,674,719).

The global prevalence rate of KOA in 2021 was 4,748.73 per 100,000 population, a 56.39% increase from 1992. Female had a prevalence rate of 5,854.31 per 100,000, higher than male’s rate of 3,650.80 per 100,000. The global incidence rate was 390.88 per 100,000 population in 2021, representing a 45.25% increase since 1992. The incidence rate was higher in female (465.41 per 100,000) than in male (316.87 per 100,000). The global YLDs rate of KOA was 152.31 per 100,000 population in 2021, a 55.71% increase compared to 1992, with female exhibiting a higher rate (186.79 per 100,000) than male (118.07 per 100,000) (see Supplementary Table 1).

In 2021, the prevalence of KOA in China was 109,575,472 cases, representing a 157.15% increase compared to 1992. The prevalence was higher among female, with 70,294,936 cases, compared to 39,280,537 cases in male. The incidence of KOA in China in 2021 was 8,512,397 cases, marking a 123.45% increase from 1992. Female also had a higher incidence, with 5,327,731 cases, compared to 3,184,666 cases in male. The YLDs due to KOA in China reached 3,554,153 person-years in 2021, a 155.29% increase since 1992. YLDs were higher in female (2,269,291) than in male (1,284,863).

The prevalence rate of KOA in China in 2021 was 7,701.69 per 100,000 population, an increase of 118.02% compared to 1992. Female had a higher prevalence rate of 10,119.58 per 100,000, while male had a rate of 5,394.91 per 100,000. The incidence rate was 598.31 per 100,000 population, 89.45% higher than in 1992, with female exhibiting a higher rate (766.97 per 100,000) compared to male (437.39 per 100,000). The YLDs rate of KOA in China was 249.81 per 100,000 population in 2021, a 116.44% increase from 1992. Female had a higher YLDs rate (326.68 per 100,000) than male (176.47 per 100,000) (Table 1).

**Table 1:** Description analysis of the burden of KOA in China in 2021.

Measure	Sex	All-ages cases, 2021, n (95% UI)	All-ages rates per 100,000 people, 2021, (95% UI)	Age-standardized rates per 100,000 people, 2021, (95% UI)	All-ages cases changes, 1992–2021, (%)	All-ages rates changes, 1992–2021, (%)	Age-standardized rates changes, 1992–2021, (%)
Prevalence	Male	39280536.59 (33096647.86, 45,575,847)	5394.91 (4545.6, 6259.53)	3661.85 (3106.11, 4228.87)	147.86	112.16	8.61
	Female	70294935.85 (59713904.72, 80953334.68)	10119.58 (8596.35, 11653.96)	6302.93 (5378.56, 7213.7)	162.65	120.44	9.42
	Both	109575472.44 (92723350.57, 126639048.86)	7701.69 (6517.21, 8901.03)	5016.52 (4265.22, 5758.38)	157.15	118.02	9.34
Incidence	Male	3184665.57 (2707999.69, 3707256.25)	437.39 (371.93, 509.17)	304.92 (261.11, 351.75)	116.54	85.35	8.77
	Female	5327731.16 (4573927.36, 6146551.04)	766.97 (658.46, 884.85)	508.53 (436.6, 583.43)	127.8	91.19	8.63
	Both	8512396.73 (7279973.65, 9840885.49)	598.31 (511.68, 691.68)	406.42 (348.7, 467.23)	123.45	89.45	9.22
YLDs (Years Lived with Disability)	Male	1284862.58 (614830.46, 2467313.99)	176.47 (84.44, 338.87)	119.45 (57.51, 229.15)	145.78	110.38	8.27
	Female	2269290.85 (1097550.01, 4397449.61)	326.68 (158, 633.05)	203.49 (98.22, 395.51)	161	119.06	9.25
	Both	3554153.43 (1715777.15, 6842993.79)	249.81 (120.6, 480.97)	162.44 (78.35, 314.13)	155.29	116.44	9.13

From 1992 to 2021, there was a rising global trend in Age-Standardized Prevalence Rate (ASPR), Age-Standardized Incidence Rate (ASIR), and Age-Standardized YLDs Rate (ASYR) of KOA (see Supplementary Table 1). Similarly, the ASPR, ASIR, and ASYR of KOA in China exhibited an increasing trend (Table 1). Notably, throughout 1992–2021, the ASPR, ASIR, and ASYR of KOA in China remained consistently higher than the global averages (Supplementary Figure 1).

**Figure 1:**
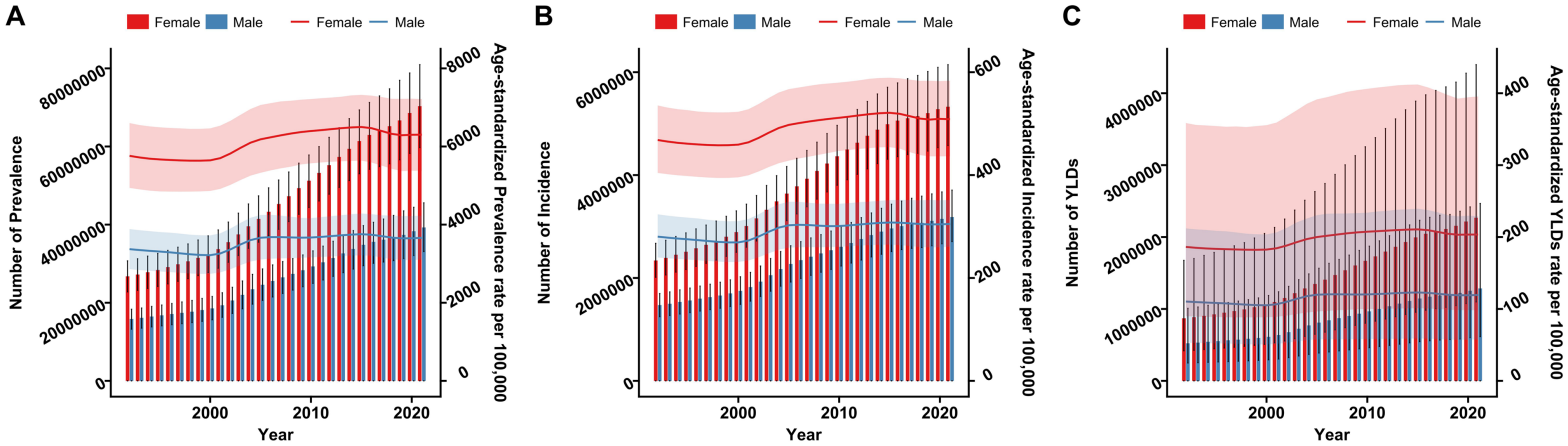
Trends in the all-age cases and age-standardized prevalence, incidence, and YLDs rates of KOA by sex from 1992 to 2021. **(A)** Prevalence number and rate. **(B)** Incidence number and rate. **(C)** YLDs number and rate.

Figure 1 illustrates the sex-specific, all-age numbers and age-standardized rates for KOA in terms of prevalence, incidence, and YLDs in China from 1992 to 2021.The number of cases, incidence, and YLDs for KOA have increased year by year from 1992 to 2021. The ASPR, ASIR, and ASYR for KOA showed an overall increasing trend amid fluctuations over the calendar years. From 1992 to 2021 in China, the ASPR, ASIR, and ASYR for KOA were consistently higher in female than in male.

### 3.2 Disease burden of KOA by gender and age in China in 2021

**Figure 2:**
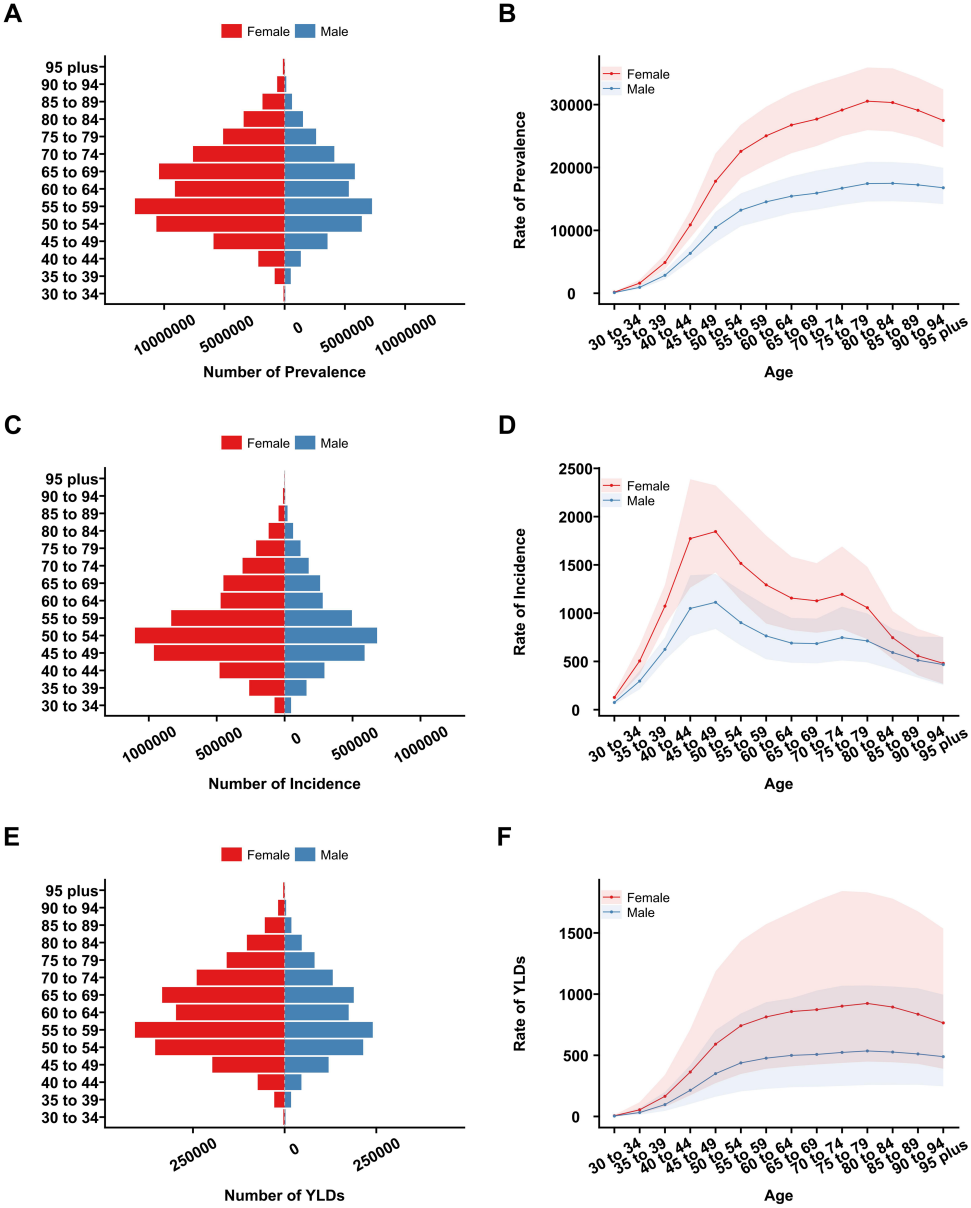
Number and rates of prevalence, incidence, and YLDs due to KOA among different age groups in China in 2021. **(A)** Prevalence number. **(B)** Prevalence rate. **(C)** Incidence number. **(D)** Incidence rate. **(E)** YLDs number. **(F)** YLDs rate.

Figure 2 displays the following for different age and sex groups in China, 2021: the number of prevalent KOA cases (panel A), the number of incident KOA cases (panel C), the number of YLDs (panel E), prevalence rates (panel B), incidence rates (panel D), and YLDs rates (panel F). The prevalence of KOA cases escalated with age, reaching a peak in the 55–59-year age group, and subsequently decreased. Similarly, the number of incident KOA cases rose until the 50–54-year age group before decreasing. The prevalence rate of KOA rose with advancing age, achieving its maximum in both sexes at ages 80–84 years. Similarly, the incidence rate increased with age, attaining its apex at ages 50–54 years for both genders. Females showed higher rates of prevalence, incidence, and YLDs than males throughout the various age ranges. The pattern observed in YLDs closely mirrored that of prevalence.

### 3.3 Trends in prevalence, incidence, and YLDs rates based on joinpoint regression analysis

**Figure 3:**
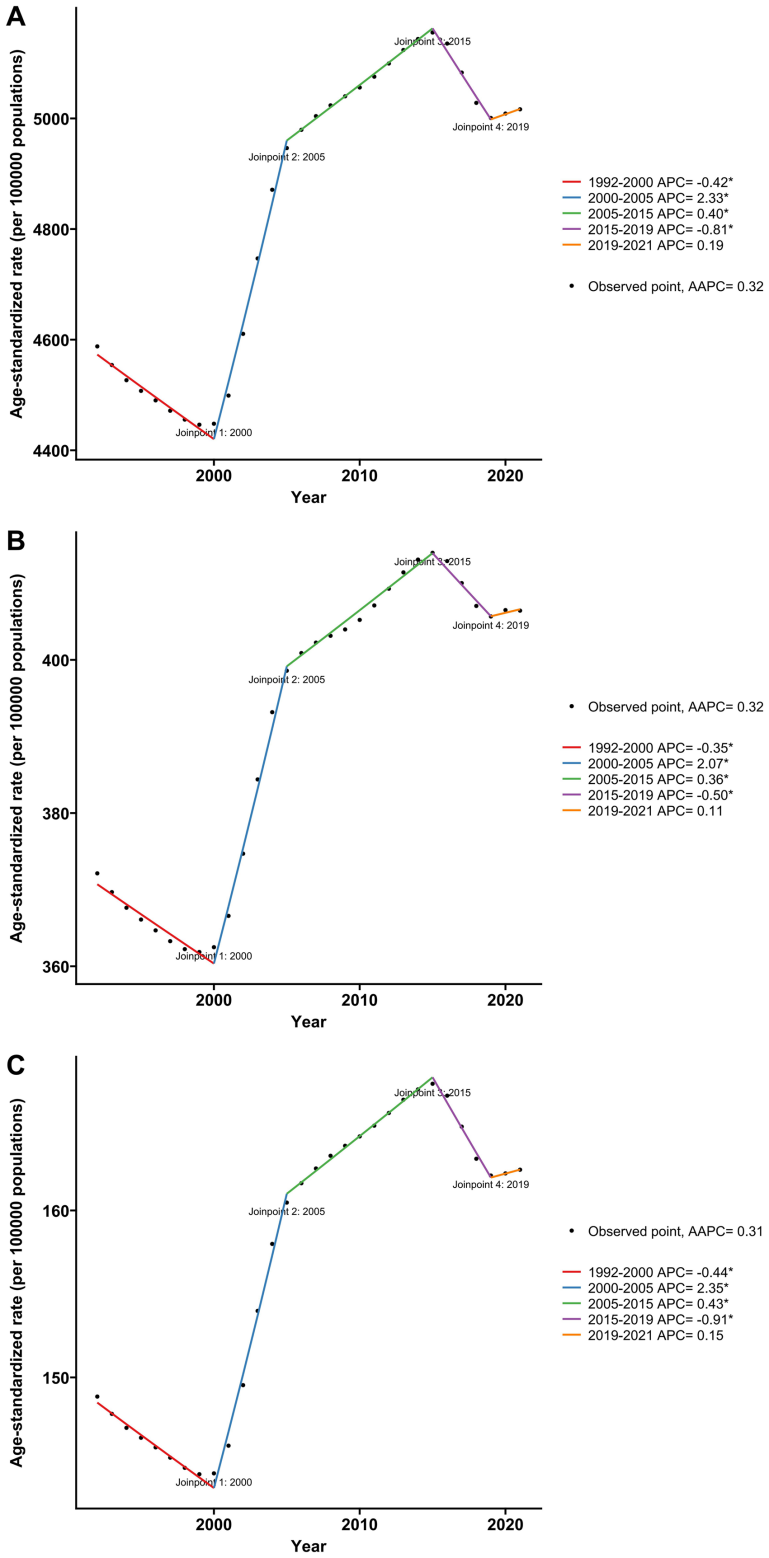
Joinpoint regression analysis of age-standardized rate for KOA in China from 1992 to 2021. **(A)** Age-standardized prevalence rate. **(B)** Age-standardized incidence rate. **(C)** Age-standardized YLDs rate.

Figure 3 presents the joinpoint regression analysis for ASPR, ASIR, and ASYR for KOA in China spanning 1992 to 2021.We observed that from 1992 to 2000, the ASPR exhibited a slight decreasing trend (APC = −0.42). From 2000 to 2005, there was a significant increase in ASPR (APC = 2.33), followed by a modest rise from 2005 to 2015 (APC = 0.40). Between 2015 and 2019, the prevalence showed a slight decreasing trend again (APC = −0.81), and from 2019 to 2021, it exhibited a slight increasing trend (APC = 0.19). The trends in ASIR and ASYR were similar to those observed for ASPR.

### 3.4 Decomposition analysis

**Figure 4:**
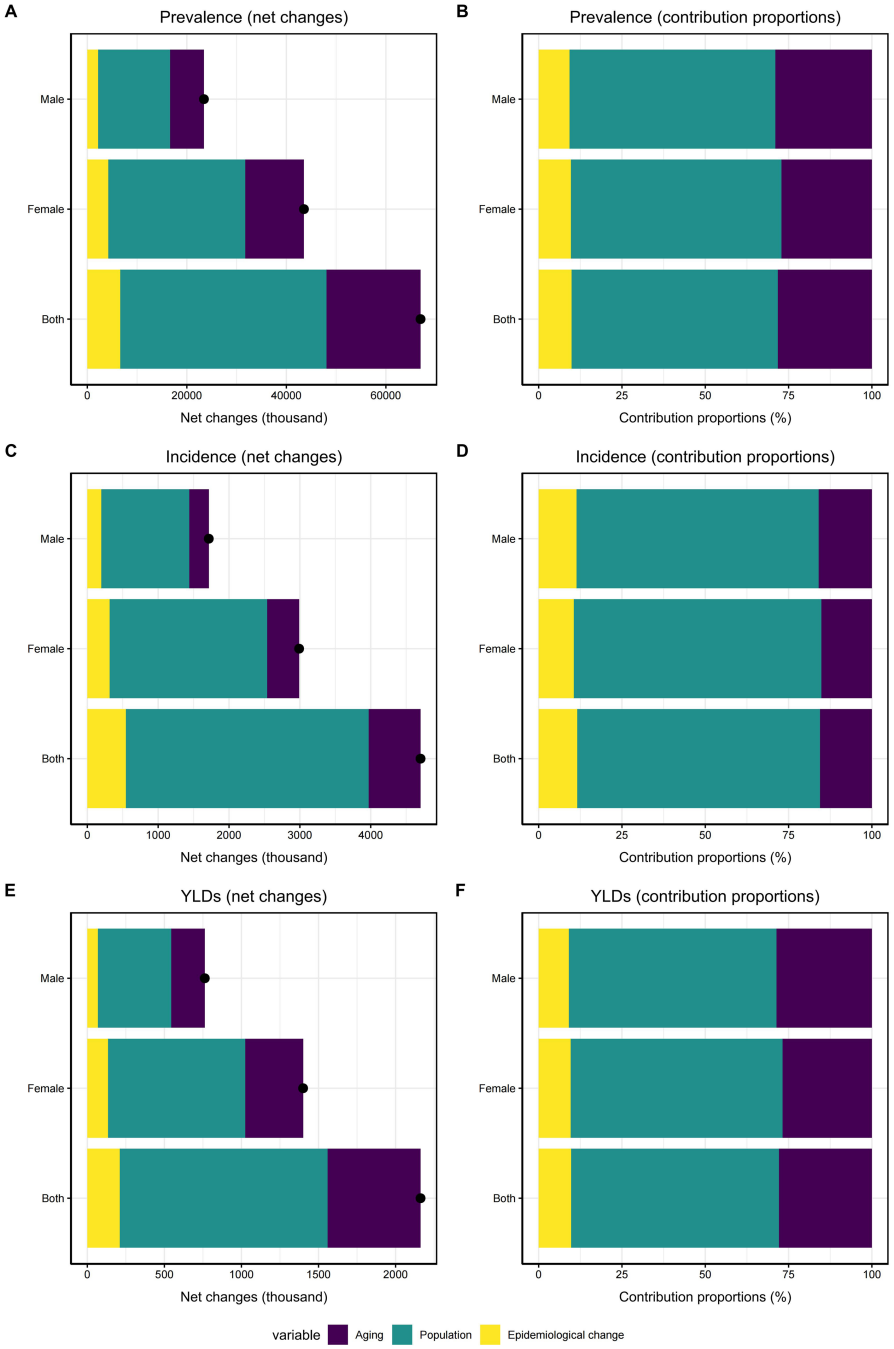
Decomposition analysis of KOA in China from 1992 to 2021. **(A)** Changes in KOA prevalence according to population growth, aging, and epidemiological change. **(B)** Proportion of the population-level determinants attributed to changes in KOA prevalence. **(C)** Changes in KOA incidence according to population growth, aging, and epidemiological change. **(D)** Proportion of the population-level determinants attributed to changes in KOA incidence. **(E)** Changes in KOA YLDs according to population growth, aging, and epidemiological change. **(F)** Proportion of the population-level determinants attributed to changes in KOA YLDs.

Our study examined the effects of epidemiological trends, population aging, and population growth on the KOA burden in China, as illustrated in Figure 4. Decomposition analysis indicated that population growth was the main driver of the rise in KOA prevalence over the past 30 years, with females contributing more significantly (63.18%) than males (61.72%). Specifically, 63.18% (approximately 27,504,277) of the total increase in prevalent cases among female and 61.72% (approximately 14,462,452) among male were attributable to population growth. The second most influential positive contributor was aging, where male demonstrated a greater proportion of impact compared to female (29.01% VS 27.17%). Additionally, the contribution of epidemiological changes was positive, accounting for 9.27% in male and 9.64% in female. The patterns observed in incidence and YLDs closely mirrored those of prevalence.

### 3.5 Age–period–cohort analysis for KOA prevalence, incidence, and YLDs rates in China

**Figure 5:**
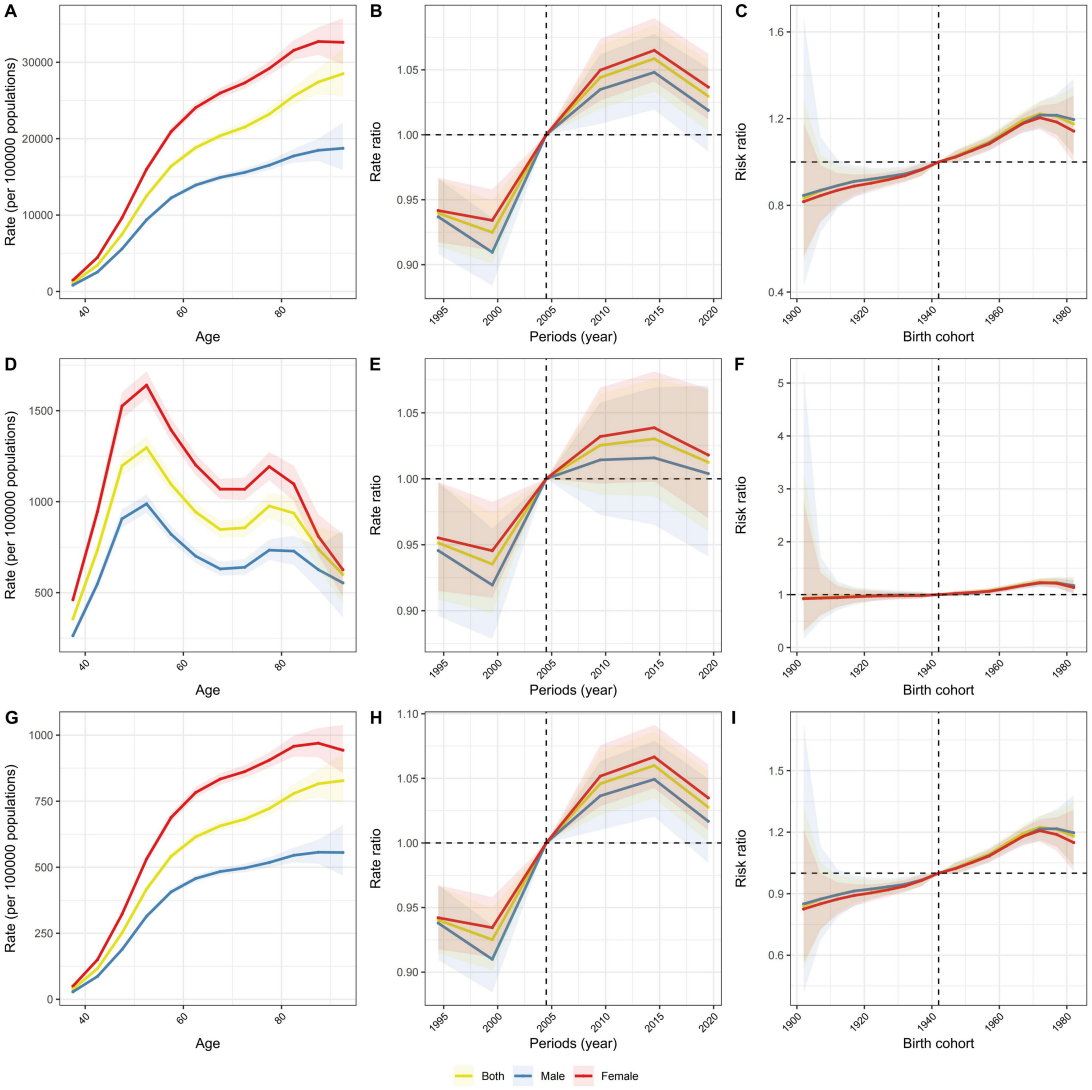
Age, period and cohort effects on KOA prevalence, incidence, and YLDs in China. **(A)** The longitudinal age curves of KOA prevalence by gender. **(B)** The period RRs of KOA prevalence by gender. **(C)** The cohort RRs of KOA prevalence by gender. **(D)** The longitudinal age curves of KOA incidence by gender. **(E)** The period RRs of KOA incidence by gender. **(F)** The cohort RRs of KOA incidence by gender. **(G)** The longitudinal age curves of KOA YLDs by gender. **(H)** The period RRs of KOA YLDs by gender. **(I)** The cohort RRs of KOA YLDs by gender.

Controlling for cohort and period effects revealed distinct trends (Figure 5): the prevalence and YLDs rates of KOA in China increased with age (panel A, G), whereas the incidence rate initially rose and then declined with advancing age (panel D). Over the last three decades, the period relative risk (RR) for incidence, prevalence, and YLDs of KOA consistently escalated (panel B, E, H). Regarding cohort effects, the incidence RR of KOA remained relatively stable, oscillating around RR = 1 (panel F). Conversely, noticeable increases were detected in the cohort RRs for both prevalence and YLDs rates among individuals born between 1902 and 1982 (panel C, I).

### 3.6 Projections of KOA YLDs rates and number for the next 9 years

**Figure 6:**
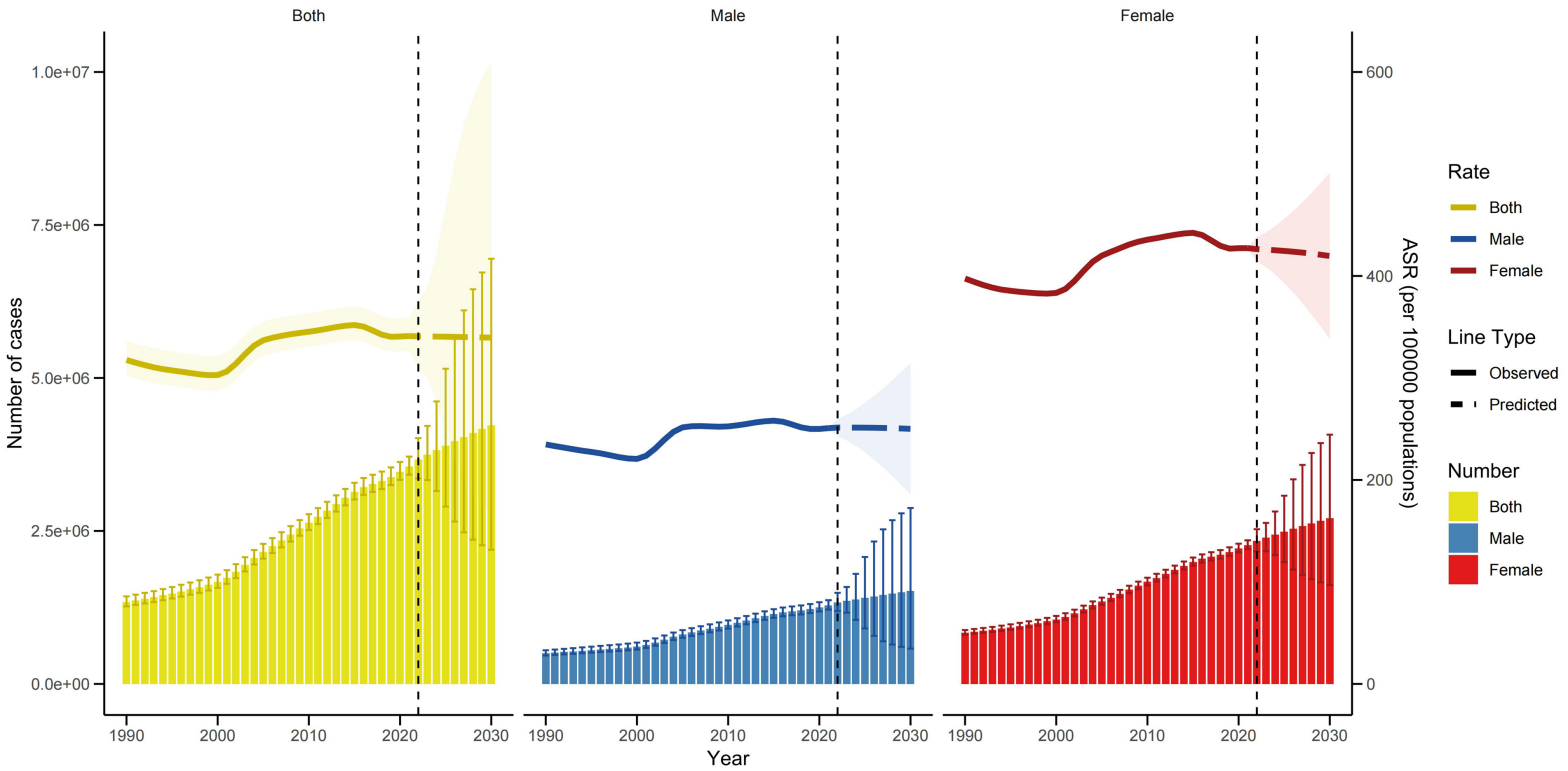
Trends in the ASYR and number of YLDs by sex in China using BAPC.

Figure 6 displays the trend in the ASYR of KOA in China from 1992 to 2030 as predicted by the BAPC model. The overall ASYR peaks in 2015 and then slightly declines, potentially reaching approximately 339.6 per 100,000 people by 2030. From 1992 to 2030, the ASYR for females shows an upward trend before slightly decreasing, potentially reaching around 419.6 per 100,000 people by 2030. The ASYR for males also exhibits a slight increase over this period and is projected to reach approximately 250.1 per 100,000 people by 2030.

Figure 6 also illustrates the observed and projected trends in the number of KOA YLDs in China from 1992 to 2030 according to the BAPC model. The total number of KOA YLDs is expected to continue rising, peaking in 2030 at approximately 4,227,552. The number of KOA YLDs among females is projected to significantly increase from 1992 to 2030, reaching approximately 2,708,657 by 2030. Similarly, the number of KOA YLDs among males shows an upward trend, potentially reaching around 1,518,895 by 2030.

**Figure 7:**
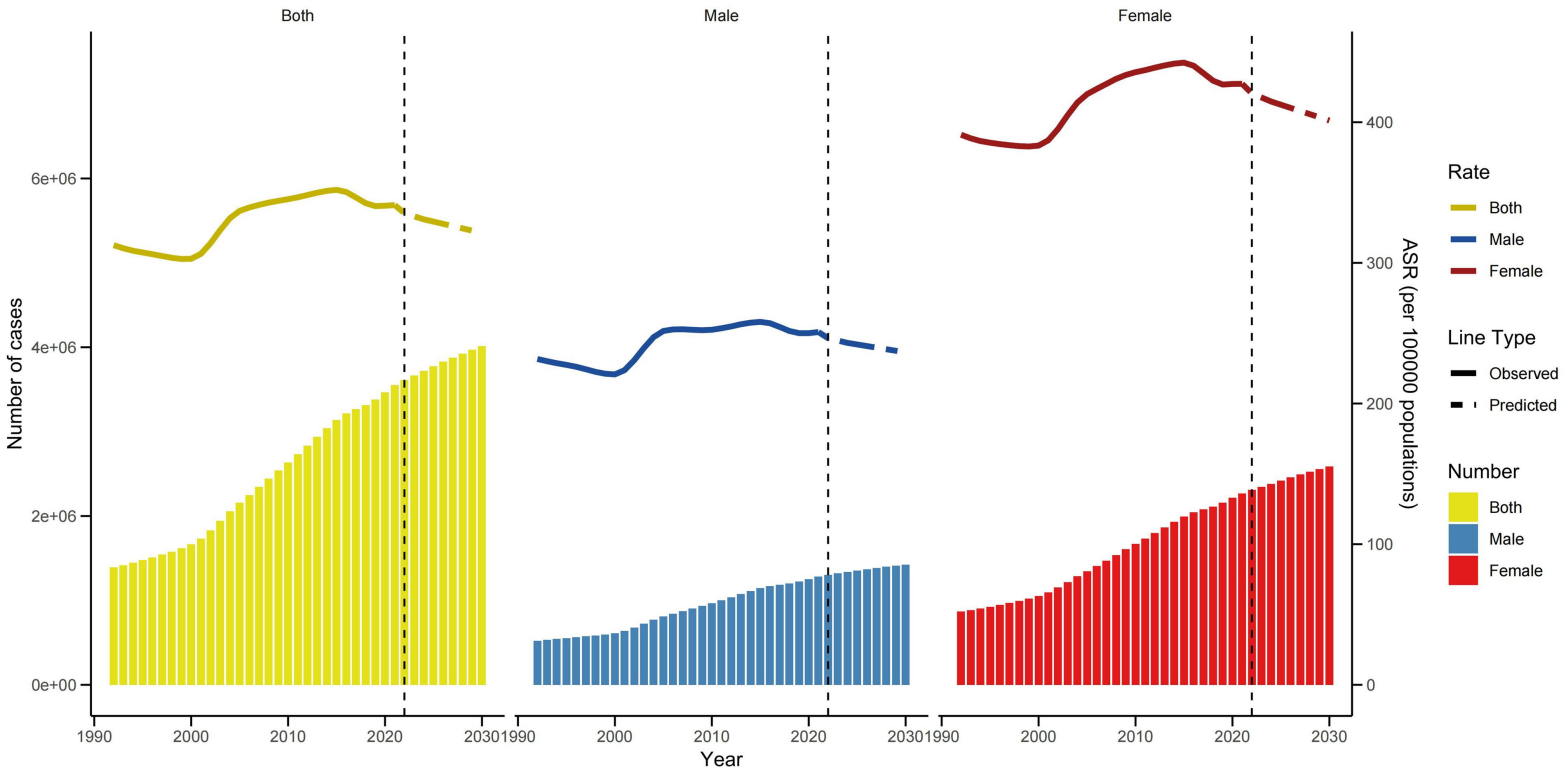
Trends in the ASYR and number of YLDs by sex in China using nordpred.

For a sensitivity analysis, the Nordpred model was employed to forecast the future burden of KOA-related YLDs in China. The results indicate a continuous downward trend in the ASYR of KOA from 2022 to 2030, potentially reaching approximately 321.3 per 100,000 people by 2030(Figure 7). Despite the decline in ASYR, the total number of KOA YLDs is projected to increase to approximately 4,011,581 by 2030. Specifically, the number of KOA YLDs among females shows an upward trend, potentially reaching approximately 2,587,508 by 2030, while the number among males is projected to rise to approximately 1,424,073 by 2030. These findings suggest that although the ASYR is decreasing, the absolute burden of KOA-related YLDs is increasing, likely due to population growth and aging.

### 3.7 Trends of associated risk factors of KOA from 1992 to 2021

**Figure 8:**
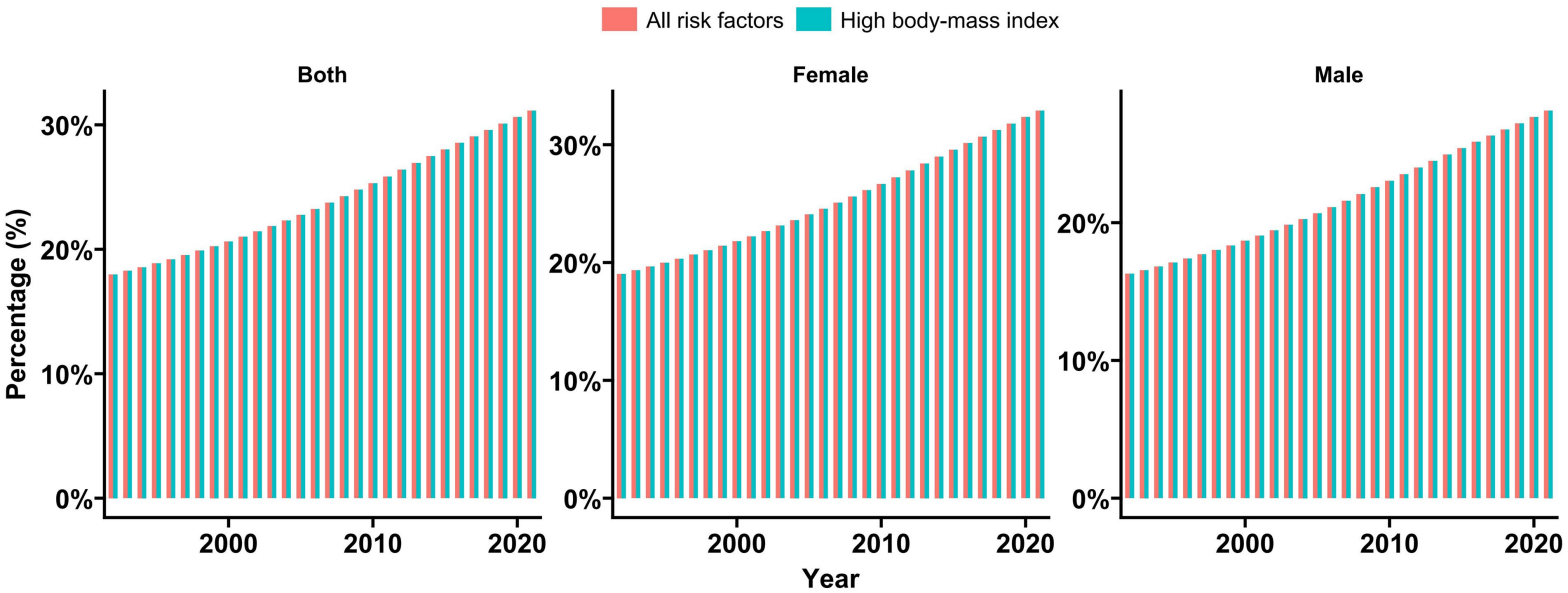
Population attributable proportion (PAF, %) of associated risk factors for osteoarthritis YLDs from 1992 to 2021 GBD 2021 identified high body mass index as risk factors for KOA YLDs.

In China, the population attributable fraction (PAF) of high BMI for KOA increased steadily from 1992 to 2021, rising from 18 to 31% during this period (Figure 8). The PAF for high BMI in females demonstrated a consistent upward trend and was always higher than that in males from 1992 to 2021.

## 4 Discussion

KOA is one of the most prevalent musculoskeletal disorders in clinical practice. This study provides the first comprehensive analysis of the burden of KOA in China and globally from 1992 to 2021, while also predicting trends for the next 9 years. The results show a significant rise in KOA incidence both in China and worldwide. In China, the number of KOA patients has reached approximately 109.6 million, marking a 157.15% increase since 1992. Globally, the number of KOA patients is estimated at 374.7 million, reflecting a 124.51% increase over the same period. Notably, the prevalence of KOA in China is rising at a rate higher than the global average. These findings are consistent with previous research (32, 33), confirming that KOA represents a substantial and growing disease burden both in China and worldwide. This highlights the urgent need for continued attention to KOA as a major public health issue, as well as the critical necessity for improved prevention strategies and treatment options.

This study examined the influence of sex on the disease burden of KOA in China. The findings indicate that the incidence, prevalence, and YLDs estimates are higher in female than in male, consistent with global studies. This confirms that sex is a significant risk factor for KOA (34). The observed differences in KOA prevalence between sexes can be attributed to several factors. First, male generally possess stronger muscles and ligaments compared to female enhancing their ability to stabilize the knee joint. Research indicates that greater muscle mass and cross-sectional area in male may contribute to a reduced risk of joint degeneration (35). In contrast, female exhibit distinct muscle engagement patterns that could adversely affect knee stability during physical activities, increasing the likelihood of joint stress (35). Additionally, anatomical differences such as a wider pelvis and narrower stride length may elevate the load on female’s knee joints (36). Moreover, the decline in estrogen levels during menopause is closely associated with the onset of KOA. Studies show that estrogen deficiency can lead to alterations in synovial tissue and cartilage, bone loss, and changes in joint function, thereby elevating the risk of KOA (37, 38). Therefore, it is essential to develop sex-specific prevention strategies and policies to address these disparities.

Additionally, this study found that the prevalence of KOA varies across different age groups, with a notable increase as age advances. The highest prevalence of KOA is observed in individuals aged 50 to 60 years, aligning with previous research findings (17). Moreover, studies have demonstrated that the risk of moderate to severe KOA is 3.56 times higher in individuals aged 65 years and older compared to younger populations (39). With advancing age, the wear and tear of articular cartilage, along with the formation of osteophytes (bone spurs), can alter the joint mechanics of the knee, leading to a decline in joint function (40). Concurrently, muscle atrophy and reduced muscle mass in older adult patients contribute to knee instability, exacerbating joint pain and dysfunction (41, 42). Furthermore, aging is characterized as a chronic inflammatory process closely associated with inflammatory factors such as tumor necrosis factor-*α* (TNF-α) and interleukin-1 (IL-1) (43), which have been shown to accelerate the onset and progression of KOA (44). It is noteworthy that the increasing aging population in China and worldwide correlates with a rising number of KOA patients. Therefore, addressing the impact of an aging population on the healthcare system, particularly concerning KOA management and treatment, presents a significant challenge.

It is important to note that the BMI is a significant risk factor for KOA. Previous studies have investigated the disease burden of KOA in China associated with elevated BMI, and the findings of this study align with those earlier investigations (20, 32). A population-based study conducted in South Korea also revealed a positive correlation between the incidence of KOA and BMI (45). Obesity contributes to increased joint wear and inflammatory responses, which can result in diminished knee joint function and heightened pain. Individuals with obesity exert greater mechanical loads on the knee joint during physical activities, compressing the meniscus, altering knee joint mechanics, and elevating the risk of joint deterioration (46, 47). Furthermore, adipose tissue secretes various inflammatory factors and adipokines that can exacerbate joint inflammation and cartilage degeneration. Research indicates that individuals with KOA have elevated serum leptin levels (48), and leptin is capable of promoting collagen production and ossification in bones, as well as stimulating osteoblast proliferation, thereby playing a role in joint inflammatory responses (49, 50).

A randomized controlled trial demonstrates that dietary management, combined with appropriate physical exercise—specifically aerobic walking and strength training—can significantly reduce BMI in patients with KOA, which also lowers plasma interleukin-6 (IL-6) levels, alleviating pain and enhancing the patients’ functional capabilities (51). Engaging in proper aerobic exercise has been shown to decrease the release of inflammatory cytokines and promote cartilage metabolism (52, 53). Meanwhile, strength training can improve quadriceps strength and enhance knee joint stability, thereby preventing the onset of KOA and mitigating its progression (54, 55). Consequently, the integration of aerobic and strength training is more effective in reducing weight among overweight KOA patients and improving their clinical symptoms (56). Additionally, the importance of dietary modifications cannot be overlooked. Research indicates that a high-protein diet can facilitate weight loss while minimizing muscle loss and enhancing joint function in KOA patients (57, 58). Therefore, it is essential for individuals at risk of KOA, as well as those already affected, to engage in appropriate exercise and dietary cooperation.

In addition, we used the BAPC model to predict the ASYR of KOA in 2030. The findings indicate that KOA will continue to impose a significant medical burden in the future. Consequently, it is essential to develop reasonable and feasible policies based on the identified risk factors associated with KOA. Weight loss has been demonstrated to effectively alleviate the symptoms of KOA, including appropriate physical exercise and a proper diet (59, 60). Furthermore, the adverse effects of sex-specific factors must be taken into account; specifically, addressing KOA resulting from postmenopausal hormonal changes, modifying unhealthy lifestyle habits, and prioritizing early diagnosis of KOA are crucial. Although age remains an immutable risk factor for KOA, ensuring timely access to medical services for older adult patients is imperative. Additionally, the importance of physical exercise should be emphasized, and special attention should be directed toward the mental health issues faced by middle-aged and older adult patients (9, 61). Therefore, attention should be focused on the disease burden caused by KOA, with the aim of reducing patients’ pain and improving their quality of life through effective policy formulation and implementation.

This study has several limitations. First, it focuses on the burden of KOA in China without analyzing the incidence characteristics across individual provinces. Second, although a brief analysis of global KOA was conducted, the regional characteristics of KOA incidence worldwide were not thoroughly explored. Third, the accuracy and completeness of GBD estimates may be constrained by the data reporting systems and collection methods used by individual countries, especially in low- and middle-income nations (23). Future research should aim to address these limitations by incorporating detailed provincial data and conducting comprehensive regional analyses globally.

## 5 Conclusion

This study found that the disease burden of KOA in China is substantial, particularly affecting female and older adults. It is essential to prioritize early diagnosis, implement effective screening measures, and promote comprehensive health education to mitigate this burden. Moreover, public health policies should be tailored to the specific characteristics of the population, focusing on high-risk groups identified in the study. Strengthening healthcare services for older adults, especially women, necessitates policy support and the strategic allocation of medical resources to ensure effective management and treatment of KOA.

## Data Availability

Publicly available datasets were analyzed in this study. This data can be found here: https://vizhub.healthdata.org/gbd-results/.

## Footnotes

^1^ https://vizhub.healthdata.org/gbd-results/
