# Supplementary material for: Burden of knee osteoarthritis in China and globally from 1992 to 2021, and projections to 2030: a systematic analysis from the Global Burden of Disease Study 2021
Source: Front Public Health. 2025 Apr 14;13:1543180. doi: 10.3389/fpubh.2025.1543180 (PMC12034567; doi:10.3389/fpubh.2025.1543180)
Supplement: Supplementary file 2 [file Presentation_1.pdf]

Supplementary Material

Case definition

Osteoarthritis (OA) is the most common form of arthritis, involving chronic inflammation, breakdown, and structural changes of whole joints. The osteoarthritis (OA) reference case definition is symptomatic osteoarthritis radiologically confirmed as Kellgren-Lawrence grade 2–4. Grade 2 symptomatic requires one defined osteophyte in the affected joint and pain for at least one month out of the last 12. Grade 3–4 symptomatic requires osteophytes and joint space narrowing in the affected joint with deformity also present for grade 4, and pain for at least one month out of the last 12 months. ICD-10 code for OA of the knee is M17.

Retrieval strategy

The retrieval strategy for the GBD 2021 is as follows: "GBD estimate": cause of death or injury; "Measure": YLDs, incidence, prevalence; "Metric": number, percentage, rate; "Cause": osteoarthritis knee; "Location": China, Global; "Age": all ages, age-standardized, 30 years to >95 years; "Sex": both, female, male; "Year": from 1992 to 2021.

The retrieval strategy for the GBD 2021 is as follows: "GBD estimate": Risk factor; "Measure": YLDs; "Metric": percentage; "Risk": All risk factors, High body-mass index; "Cause": osteoarthritis knee; "Location": China; "Age": age-standardized; "Sex": both, female, male; "Year": from 1992 to 2021.

Furthermore, in the GBD database, we selected the China field, which includes the Hong Kong Special Administrative Region and the Macao Special Administrative Region, but did not select Taiwan (Province of China).
