# Supplementary figures and images for: Burden of knee osteoarthritis in China and globally from 1992 to 2021, and projections to 2030: a systematic analysis from the Global Burden of Disease Study 2021
Source: Front Public Health. 2025 Apr 14;13:1543180. doi: 10.3389/fpubh.2025.1543180 (PMC12034567; doi:10.3389/fpubh.2025.1543180)

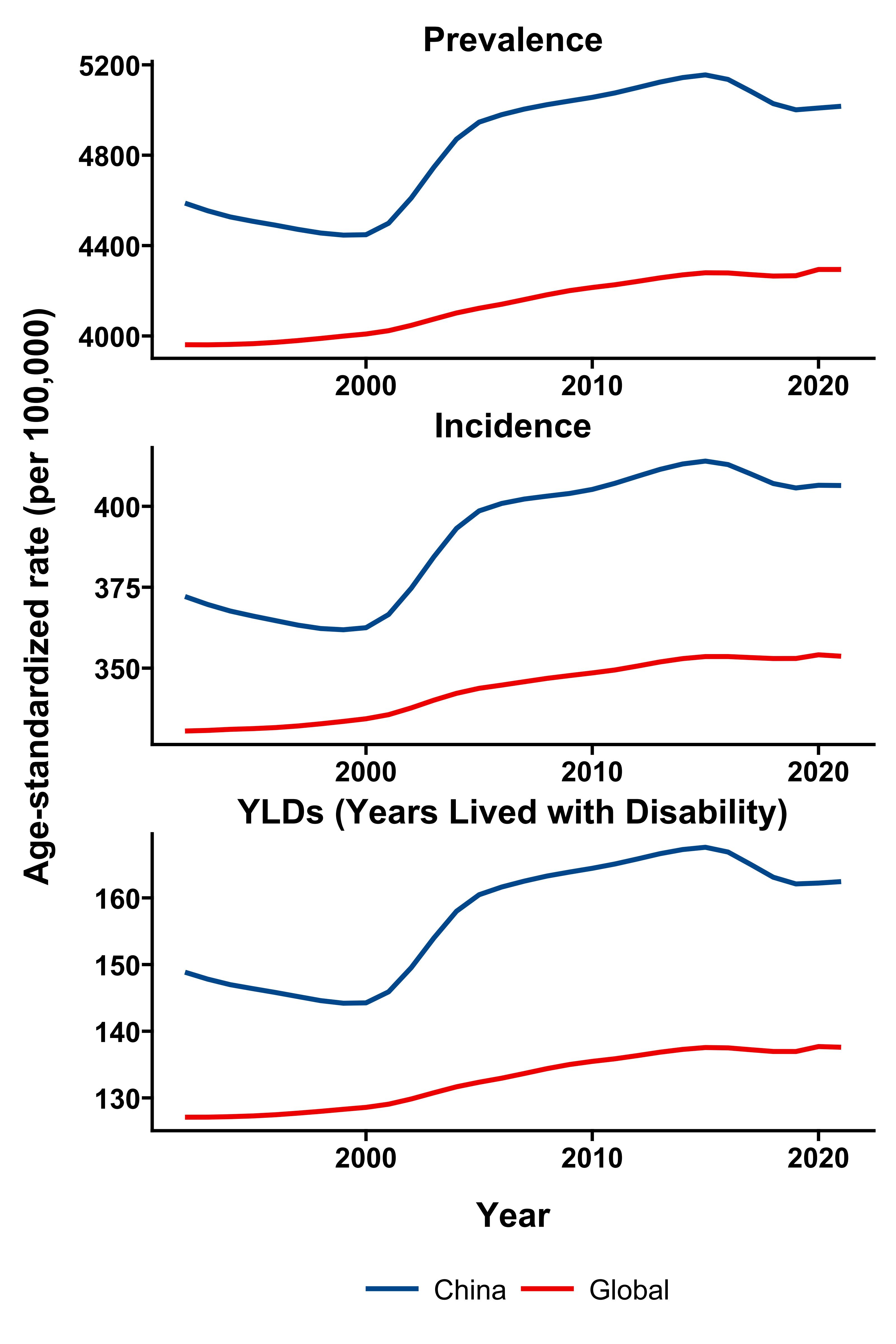

Supplement: Supplementary file 3 [file Image_1.jpeg]
